# Cardinality Balanced Multi-Target Multi-Bernoulli Filter with Error Compensation

**DOI:** 10.3390/s16091399

**Published:** 2016-08-31

**Authors:** Xiangyu He, Guixi Liu

**Affiliations:** 1 School of Mechano-electronic Engineering, Xidian University, Xi’an 710071, China; hexyming@163.com; 2 School of Physics and Electronic Information, Luoyang Normal University, Luoyang 471934, China

**Keywords:** error compensation, multi-target multi-Bernoulli filter, multi-target tracking, random finite set

## Abstract

The cardinality balanced multi-target multi-Bernoulli (CBMeMBer) filter developed recently has been proved an effective multi-target tracking (MTT) algorithm based on the random finite set (RFS) theory, and it can jointly estimate the number of targets and their states from a sequence of sensor measurement sets. However, because of the existence of systematic errors in sensor measurements, the CBMeMBer filter can easily produce different levels of performance degradation. In this paper, an extended CBMeMBer filter, in which the joint probability density function of target state and systematic error is recursively estimated, is proposed to address the MTT problem based on the sensor measurements with systematic errors. In addition, an analytic implementation of the extended CBMeMBer filter is also presented for linear Gaussian models. Simulation results confirm that the proposed algorithm can track multiple targets with better performance.

## 1. Introduction

Recently, the random finite set (RFS) theory [1] has provided an elegant formulation for the multi-target tracking (MTT) problem and has already gained substantial interest. The probability hypothesis density (PHD) multi-target filter [2] is an effective approach for tracking multiple targets based on the RFS theory, as it can simultaneously estimate the number and the state of targets without the measurement-to-track association used in the traditional MTT approaches [3,4,5,6]. The PHD filter needs to calculate multiple integrals, and the integrals might be also intractable in many cases of interest. In order to overcome the inherent intractability of the PHD filter, two major implementations for the PHD filter have been developed. One is known as the sequential Monte Carlo (SMC)-PHD filter or particle PHD filter [7,8] and the other is known as the Gaussian mixture (GM)-PHD filter [9,10]. The particle PHD filter uses a large number of particles to approximate the PHD distribution, while the GM-PHD filter estimates the PHD distribution as a mixture of Gaussian densities. Convergence results for the particle PHD filter and GM-PHD filter have been given in [11,12], respectively. The resulting PHD filter subsequently became a very popular multi-target tracking method with applications in sonar image tracking [13], video target tracking [14,15], vehicle cooperative localization [16], etc.

The PHD filter may produce unreliable estimates of the number of targets due to the Poisson assumption for the target number distribution. Subsequently, the cardinalized probability hypothesis density (CPHD) filter [17] was established to overcome the problem present in the PHD filter. The Gaussian mixture CPHD (GM-CPHD) filter [18] provides a closed-form solution to the CPHD filter for tracking multiple targets in practice. Compared with GM-PHD filter, the GM-CPHD filter provides more accurate estimates of target number but with higher computational cost, as the filter recursion equations for the target number distribution and intensity function are coupled.

The multi-target multi-Bernoulli (MeMBer) recursion [19], which propagates the multi-target posterior density approximately, is another approximation to the multi-target Bayes filter using multi-Bernoulli RFS. However, it has been analyzed that the MeMBer filter overestimates the number of targets. A satisfactory solution named the cardinality-balanced MeMBer (CBMeMBer) filter has been proposed to reduce the posterior cardinality bias by modifying the measurement-updated tracks parameters [20]. Similar to the PHD filter, there are two major implementations of the CBMeMBer filter known as the particle CBMeMBer filter and the Gaussian mixture (GM-CBMeMBer) filter [20]. Moreover, the convergence results for the particle CBMeMBer filter have been given in [21]. Afterwards, the work in [22] proposes an improved MTT algorithm based on the CBMeMBer filter and variational Bayesian approximation to track multiple targets for the linear Gaussian models with unknown measurement noise variances. To track multiple maneuvering targets, two different extensions based on the CBMeMBer filter and the multi-model method have been proposed in [23,24]. Following the CBMeMBer filter in MTT scenarios, a forward-backward CBMeMBer smoothing algorithm aimed at improving the performance of the CBMeMBer-based filtering algorithms was proposed in [25]. In addition, based on the recently introduced labeled RFS formulation, the generalized labeled multi-Bernoulli (GLMB) filter [26,27] was proposed as an improved approximation of the MeMBer filter. The GLMB filter is superior to the MeMBer filter in the aspect of estimation accuracy, but with a major disadvantage of greater computational complexity than the MeMBer filter, which is at worst cubic in the number of measurements.

In this paper, we focus on the CBMeMBer filter due to its computational efficiency and satisfactory filtering accuracy compared with other RFS-based filters. As is well known, the purpose of MTT is to recursively estimate the target numbers and their states by using the cluttered measurement sets collected by sensors. In practical applications, the measurements produced by an imperfect sensor are usually characterized by both the random noise and systematic errors or biases. This implies that the measurement model is influenced by a bias vector and the sensor measurements are biased. Hence, if the bias is not incorporated in the measurement model, the performance of the filter will degrade. The GM-CBMeMBer filter, whose prediction and update steps for each hypothesized track are performed by using the Kalman filtering technique, is a closed solution to the CBMeMBer filter for linear Gaussian models. However, for the standard GM-CBMeMBer filter, the systematic errors in sensor measurements are not considered. The existence of systematic errors in sensor measurements will affect the accuracy of target position estimations. That is, the position estimations will be biased. In addition, the existence probabilities of new targets at the time steps where new targets appear depend on the measurement-updated tracks, while the existence probabilities of surviving targets depend on the legacy tracks. Hence, at the time steps where the new targets appear, the existence of systematic errors might lead to problems such as the target number being underestimated and the position estimations of the new targets being lost.

In this paper, an extended CBMeMBer filter is proposed to address the problem of MTT with systematic errors. By introducing the joint probability density function of the target state and systematic error, the proposed filter can be derived from modifying the CBMeMBer recursion equations directly. In addition, the analytic implementation of the extended CBMeMBer filter is also derived by using the bias measurement models and the linear Gaussian assumptions on target models. Simulation results verify that the proposed algorithm outperforms GM-CBMeMBer filter in both the aspects of target state estimation and target number estimation by using the biased measurements.

The rest of this paper is organized as follows. In Section 2 an extended CBMeMBer recursion is provided. The analytic implementation of the extended CBMeMBer recursion is elaborated in Section 3. In Section 4, the simulated results are presented and discussed. Finally, some meaningful conclusions are given in Section 5.

## 2. Extended CBMeMBer Filter

In MTT problems, the numbers of targets and measurements are time-variant due to targets and clutter appearing and disappearing. The linear Gaussian dynamic and bias measurement models that each target in two-dimensional plane follows can be written as

(1) $$x_{k}=F_{k-1}x_{k-1}+G_{k-1}q_{k-1}$$

(2) $$z_{k}=H_{k}x_{k}+b_{k}+v_{k}$$

where $x_{k}$ is the target state vector at time step k, and $F_{k-1}$ and $G_{k-1}$ are the state transition matrix and the noise input matrix, respectively. $z_{k}$ and $H_{k}$ are the measurement vector and the observation matrix. The state noise $q_{k-1}$ is assumed to be zero-mean white Gaussian noise with covariance $Q_{k-1}$, and the measurement noise $v_{k}$ is zero-mean white Gaussian noise with covariance $R_{k}$. $b_{k}$, which denotes the sensor systematic error vector.

As defined in [28], the systematic error $b_{k}$ can be modeled as a first-order Gauss–Markov process. From Equation (2), it can be seen that the existence of systematic errors in sensor measurements means that the value of $E(z_{k}-H_{k}x_{k})$ is not zero and results in damages to the performance of target tracks update. The damage of systematic error on the performance of target tracks update is different from that of random noise because it cannot be reduced by simple averaging.

To reduce the influence of systematic errors on filtering results, and for the CBMeMBer update functions to perform adequately, an extended CBMeMBer filter is proposed in this section. For the derivation of the extended CBMeMBer filter, we can treat $\left(x_{k},b_{k}\right)$ as the augmented state and express the joint probability density function of $x_{k}$ and $b_{k}$ as $p\;(x_{k},b_{k})$. The extended CBMeMBer filter is derived from substituting the augmented state model parameters into the standard CBMeMBer recursion equations. The prediction and update equations of the proposed filter derived are given in the following subsections.

### 2.1. Prediction

At time step k−1, if the joint posterior multi-target density is a multi-Bernoulli and has the form

(3) $$\pi_{k-1}(x_{k-1},b_{k-1})={\left\{(r_{k-1}^{\left(i\right)},p_{k-1}^{\left(i\right)}(x_{k-1},b_{k-1}))\right\}}_{i=1}^{M_{k-1}}$$

where $r_{k-1}^{\left(i\right)}$ is the existence probability of the *i*th hypothesized track, $p_{k-1}^{\left(i\right)}(x_{k-1},b_{k-1})$ denotes the joint probability density function of $x_{k-1}$ and $b_{k-1}$, $M_{k-1}$ is the number of hypothesized tracks.

Suppose that the target state $x_{k}$ and the systematic error $b_{k}$ are uncorrelated. Then, the predicted joint multi-target density at time step k is also a multi-Bernoulli and given by

(4) $$\pi_{k|k-1}(x_{k},b_{k})={\left\{(r_{P,k|k-1}^{\left(i\right)},p_{P,k|k-1}^{\left(i\right)}(x_{k},b_{k}))\right\}}_{i=1}^{M_{k-1}}\cup {\left\{(r_{\gamma ,k}^{\left(i\right)},p_{\gamma ,k}^{\left(i\right)}(x_{k},b_{k}))\right\}}_{i=1}^{M_{\gamma ,k}}$$

where ${\left\{(r_{\gamma ,k}^{\left(i\right)},p_{\gamma ,k}^{\left(i\right)}(x_{k},b_{k}))\right\}}_{i=1}^{M_{\gamma ,k}}$ denotes the parameter set of the multi-Bernoulli RFS of births at time step k, and

(5) $$r_{P,k|k-1}^{\left(i\right)}=r_{k-1}^{\left(i\right)}\langle p_{k-1}^{\left(i\right)}(x_{k-1},b_{k-1}),p_{S}\rangle =r_{k-1}^{\left(i\right)}\iint p_{S}p_{k-1}^{\left(i\right)}(x_{k-1},b_{k-1})dx_{k-1}db_{k-1}$$

(6) $$\begin{array}{ll} p_{P,k|k-1}^{\left(i\right)}(x_{k},b_{k}) & =\frac{\langle f_{k|k-1}(x_{k},b_{k}|x_{k-1},b_{k-1}),p_{S}p_{k-1}^{\left(i\right)}(x_{k-1},b_{k-1})\rangle }{\langle p_{k-1}^{\left(i\right)}(x_{k-1},b_{k-1}),p_{S}\rangle }\\ & =\frac{\langle f_{k|k-1}(x_{k}|x_{k-1})f_{k|k-1}(b_{k}|b_{k-1}),p_{S}p_{k-1}^{\left(i\right)}(x_{k-1},b_{k-1})\rangle }{\langle p_{k-1}^{\left(i\right)}(x_{k-1},b_{k-1}),p_{S}\rangle }\\ & =\frac{\iint p_{S}f_{k|k-1}(x_{k}|x_{k-1})f_{k|k-1}(b_{k}|b_{k-1})p_{k-1}^{\left(i\right)}(x_{k-1},b_{k-1})dx_{k-1}db_{k-1}}{\iint p_{S}p_{k-1}^{\left(i\right)}(x_{k-1},b_{k-1})dx_{k-1}db_{k-1}} \end{array}$$

where 〈⋅,⋅〉 denotes inner product, $p_{S}$ is the target survival probability, $f_{k|k-1}(b_{k}|b_{k-1})$ denotes the transition density of the systematic error, and $f_{k|k-1}(x_{k}|x_{k-1})$ is the single target transition density.

### 2.2. Update

If the predicted joint multi-target density at time step k is a multi-Bernoulli and has the form

(7) $$\pi_{k|k-1}(x_{k},b_{k})={\left\{(r_{k|k-1}^{\left(i\right)},p_{k|k-1}^{\left(i\right)}(x_{k},b_{k}))\right\}}_{i=1}^{M_{k|k-1}}$$

then, the updated joint multi-target density at time step k can be also approximated by a multi-Bernoulli as

(8) $$\pi_{k}(x_{k},b_{k})={\left\{(r_{L,k}^{\left(i\right)},p_{L,k}^{\left(i\right)}(x_{k},b_{k}))\right\}}_{i=1}^{M_{k|k-1}}\cup {\left\{(r_{U,k}(z_{k}),p_{U,k}(x_{k},b_{k};z_{k}))\right\}}_{z_{k}\in Z_{k}}$$

where

(9) $$r_{L,k}^{\left(i\right)}=r_{k|k-1}^{\left(i\right)}\frac{1-\chi_{k|k-1}^{\left(i\right)}}{1-r_{k|k-1}^{\left(i\right)}\chi_{k|k-1}^{\left(i\right)}}$$

(10) $$p_{L,k}^{\left(i\right)}(x_{k},b_{k})=\frac{1-p_{D}}{1-\chi_{k|k-1}^{\left(i\right)}}p_{k|k-1}^{\left(i\right)}(x_{k},b_{k})$$

(11) $$r_{U,k}(z_{k})=\frac{\sum_{i=1}^{M_{k|k-1}}\frac{r_{k|k-1}^{\left(i\right)}(1-r_{k|k-1}^{\left(i\right)})\psi_{k}^{\left(i\right)}(z_{k})}{\left(1-r_{k|k-1}^{\left(i\right)}\chi_{k|k-1}^{\left(i\right)}\right){}^{2}}}{\kappa_{k}(z_{k})+\sum_{i=1}^{M_{k|k-1}}\frac{r_{k|k-1}^{\left(i\right)}\psi_{k}^{\left(i\right)}(z_{k})}{1-r_{k|k-1}^{\left(i\right)}\chi_{k|k-1}^{\left(i\right)}}}$$

(12) $$p_{U,k}(x_{k},b_{k};z_{k})=\frac{\sum_{i=1}^{M_{k|k-1}}\frac{r_{k|k-1}^{\left(i\right)}}{1-r_{k|k-1}^{\left(i\right)}}p_{D}p_{k|k-1}^{\left(i\right)}(x_{k},b_{k})g_{k}(z_{k}|x_{k},b_{k})}{\sum_{i=1}^{M_{k|k-1}}\frac{r_{k|k-1}^{\left(i\right)}}{1-r_{k|k-1}^{\left(i\right)}}\psi_{k}^{\left(i\right)}(z_{k})}$$

(13) $$\begin{array}{lll} \chi_{k|k-1}^{\left(i\right)} & = & \langle p_{k|k-1}^{\left(i\right)}(x_{k},b_{k}),p_{D}\rangle \\ & = & \iint p_{D}p_{k|k-1}^{\left(i\right)}(x_{k},b_{k})dx_{k}db_{k} \end{array}$$

(14) $$\begin{array}{lll} \psi_{k}^{\left(i\right)}(z_{k}) & = & \langle g_{k}(z_{k}|x_{k},b_{k}),p_{D}p_{k|k-1}^{\left(i\right)}(x_{k},b_{k})\rangle \\ & = & \iint p_{D}p_{k|k-1}^{\left(i\right)}(x_{k},b_{k})g_{k}(z_{k}|x_{k},b_{k})dx_{k}db_{k} \end{array}$$

where $p_{D}$ is the detection probability, $z_{k}$ is the measurement set received at time step k, $g_{k}(\cdot |x,b)$ is the single-target measurement likelihood, and $\kappa_{k}(z)$ is the intensity of Poisson clutter.

From the above recursion equations, it can be seen that the extended CBMeMBer filter is generally intractable because of the existence of multiple integrals. To obtain close-form solutions, an analytic implementation of the extended CBMeMBer filter is proposed in the next section.

## 3. Analytic Implementation of the Extended CBMeMBer Filter

To facilitate the derivation of the analytic implementation, we first rewrite the linear Gaussian dynamic and bias measurement models represented by Equations (1) and (2) in the following form

(15) $$f_{k|k-1}(x_{k}|x_{k-1})=N(x_{k};F_{k-1}x_{k-1},G_{k-1}Q_{k-1}G_{k-1}^{T})$$

(16) $$g_{k}(z_{k}|x_{k},b_{k})=N(z_{k};H_{k}x_{k}+b_{k},R_{k})$$

where N(⋅;m,P) denotes a Gaussian function with mean m and covariance P.

In addition, the birth model at time step k is assumed to be a multi-Bernoulli with parameter set ${\left\{(r_{\gamma ,k}^{\left(i\right)},p_{\gamma ,k}^{\left(i\right)}(x_{k},b_{k}))\right\}}_{i=1}^{M_{\gamma ,k}}$, and the joint probability density of the *i*th birth track $p_{\gamma ,k}^{\left(i\right)}(x_{k},b_{k})$ has the form

(17) $$p_{\gamma ,k}^{\left(i\right)}(x_{k},b_{k})=\sum_{j=1}^{J_{\gamma ,k}^{\left(i\right)}}w_{\gamma ,k}^{\left(i,j\right)}N(x_{k};m_{\gamma ,k}^{\left(i,j\right)},P_{\gamma ,k}^{\left(i,j\right)})N(b_{k};{\hat{m}}_{\gamma ,k}^{\left(i,j\right)},{\hat{P}}_{\gamma ,k}^{\left(i,j\right)})$$

Finally, the systematic error $b_{k}$ is the first order Gauss–Markov process given by

(18) $$f_{k|k-1}(b_{k}|b_{k-1})=N(b_{k};b_{k-1},B_{k-1})$$

Similar to the GM-CBMeMBer filter [20], the analytic implementation of the extended CBMeMBer filter can be carried out by applying the standard results for Gaussian functions given in [29]. The following subsections show how the multi-Bernoulli joint posterior density is analytically propagated in time.

### 3.1. Prediction

Suppose that at time step k−1, the multi-Bernoulli joint posterior density $\pi_{k-1}(x_{k-1,}b_{k-1})$ has the form of Equation (3), where $p_{k-1}^{\left(i\right)}(x_{k-1},b_{k-1})$ can be expressed as

(19) $$p_{k-1}^{\left(i\right)}(x_{k-1},b_{k-1})=\sum_{j=1}^{J_{k-1}^{\left(i\right)}}w_{k-1}^{\left(i,j\right)}N(x_{k-1};m_{k-1}^{\left(i,j\right)},P_{k-1}^{\left(i,j\right)})N(b_{k-1};{\hat{m}}_{k-1}^{\left(i,j\right)},{\hat{P}}_{k-1}^{\left(i,j\right)})$$

Then, the predicted multi-target density $\pi_{k|k-1}(x_{k},b_{k})$ at time step k is the same as Equation (4), where the parameters in ${\left\{(r_{\gamma ,k}^{\left(i\right)}p_{\gamma ,k}^{\left(i\right)}(x_{k},b_{k}))\right\}}_{i=1}^{M_{\gamma ,k}}$ are given in Equation (17). In addition, substituting Equations (15), (18) and (19) into Equations (5) and (6), other parameters in $\pi_{k|k-1}(x_{k},b_{k})$ can be derived as follows

(20) $$r_{P,k|k-1}^{\left(i\right)}=p_{S}r_{k-1}^{\left(i\right)}$$

(21) $$p_{P,k|k-1}^{\left(i\right)}(x_{k},b_{k})=\sum_{j=1}^{J_{k-1}^{\left(i\right)}}w_{k|k-1}^{\left(i,j\right)}N(x_{k};m_{P,k|k-1}^{\left(i,j\right)},P_{k|k-1}^{\left(i,j\right)})N(b_{k};{\hat{m}}_{P,k|k-1}^{\left(i,j\right)},{\hat{P}}_{P,k|k-1}^{\left(i,j\right)})$$

(22) $$m_{P,k|k-1}^{\left(i,j\right)}=F_{k-1}m_{k-1}^{\left(i,j\right)}$$

(23) $$P_{P,k|k-1}^{\left(i,j\right)}=G_{k-1}Q_{k-1}G_{k-1}^{T}+F_{k-1}P_{k-1}^{\left(i,j\right)}F_{k-1}^{T}$$

(24) $${\hat{m}}_{P,k|k-1}^{\left(i,j\right)}={\hat{m}}_{k-1}^{\left(i,j\right)}$$

(25) $${\hat{P}}_{P,k|k-1}^{\left(i,j\right)}={\hat{P}}_{P,k|k-1}^{\left(i,j\right)}+B_{k-1}$$

### 3.2. Update

Suppose that at time step k, the multi-Bernoulli joint posterior density $\pi_{k|k-1}(x_{k,}b_{k})$ has the form of Equation (7), where $p_{k|k-1}^{\left(i\right)}(x_{k},b_{k})$ can be written as

(26) $$p_{k|k-1}^{\left(i\right)}(x_{k},b_{k})=\sum_{j=1}^{J_{k|k-1}^{\left(i\right)}}w_{k|k-1}^{\left(i,j\right)}N(x_{k};m_{k|k-1}^{\left(i,j\right)},P_{k|k-1}^{\left(i,j\right)})N(b_{k};{\hat{m}}_{k|k-1}^{\left(i,j\right)},{\hat{P}}_{k|k-1}^{\left(i,j\right)})$$

Then the updated joint multi-target density $\pi_{k}(x_{k},b_{k})$ at time step k is the same as Equation (8). Substituting Equations (16) and (26) into Equations (9)–(14), the derived equations for calculating the parameters in $\pi_{k}(x_{k},b_{k})$ are presented as follows

(27) $$r_{L,k}^{\left(i\right)}=r_{k|k-1}^{\left(i\right)}\frac{1-p_{D}}{1-p_{D}r_{k|k-1}^{\left(i\right)}}$$

(28) $$p_{L,k}^{\left(i\right)}(x_{k},b_{k})=p_{k|k-1}^{\left(i\right)}(x_{k},b_{k})$$

(29) $$r_{U,k}(z_{k})=\frac{\sum_{i=1}^{M_{k|k-1}}\frac{r_{k|k-1}^{\left(i\right)}(1-r_{k|k-1}^{\left(i\right)})\psi_{k}^{\left(i\right)}(z_{k})}{\left(1-p_{D}r_{k|k-1}^{\left(i\right)}\right){}^{2}}}{\kappa_{k}(z_{k})+\sum_{i=1}^{M_{k|k-1}}\frac{r_{k|k-1}^{\left(i\right)}\psi_{k}^{\left(i\right)}(z_{k})}{1-p_{D}r_{k|k-1}^{\left(i\right)}}}$$

(30) $$\psi_{k}^{\left(i\right)}(z_{k})=p_{D}\sum_{j=1}^{J_{k|k-1}^{\left(i\right)}}w_{k|k-1}^{\left(i,j\right)}N(z_{k};H_{k}m_{k|k-1}^{\left(i,j\right)}+{\hat{m}}_{k|k-1}^{\left(i,j\right)},R_{k}+{\hat{P}}_{k|k-1}^{\left(i,j\right)}+H_{k}P_{k|k-1}^{\left(i,j\right)}H_{k}^{T})$$

(31) $$p_{U,k}(x_{k},b_{k};z_{k})=\frac{\sum_{i=1}^{M_{k|k-1}}\sum_{j=1}^{J_{k|k-1}^{\left(i\right)}}w_{U,k}^{\left(i,j\right)}N(x_{k};m_{U,k}^{\left(i,j\right)},P_{U,k}^{\left(i,j\right)})N(b_{k};{\hat{m}}_{U,k}^{\left(i,j\right)},{\hat{P}}_{U,k}^{\left(i,j\right)})}{\sum_{i=1}^{M_{k|k-1}}\sum_{j=1}^{J_{k|k-1}^{\left(i\right)}}w_{U,k}^{\left(i,j\right)}}$$

(32) $$w_{U,k}^{\left(i,j\right)}=\frac{r_{k|k-1}^{\left(i\right)}}{1-r_{k|k-1}^{\left(i\right)}}\psi_{k}^{\left(i\right)}(z_{k})$$

(33) $$m_{U,k}^{\left(i,j\right)}=m_{k|k-1}^{\left(i,j\right)}+K_{U,k}^{\left(i,j\right)}(z_{k}-H_{k}m_{k|k-1}^{\left(i,j\right)}-{\hat{m}}_{k|k-1}^{\left(i,j\right)})$$

(34) $$P_{U,k}^{\left(i,j\right)}=(I-K_{U,k}^{\left(i,j\right)}H_{k})P_{k|k-1}^{\left(i,j\right)}$$

(35) $$K_{U,k}^{\left(i,j\right)}=P_{k|k-1}^{\left(i,j\right)}H_{k}^{T}\left(R_{k}+H_{k}P_{k|k-1}^{\left(i,j\right)}H_{k}^{T}\right){}^{-1}$$

(36) $${\hat{m}}_{U,k}^{\left(i,j\right)}={\hat{m}}_{k|k-1}^{\left(i,j\right)}+{\hat{P}}_{k|k-1}^{\left(i,j\right)}\left(R_{k}+{\hat{P}}_{k|k-1}^{\left(i,j\right)}+H_{k}P_{k|k-1}^{\left(i,j\right)}H_{k}^{T}\right){}^{-1}(z_{k}-H_{k}m_{k|k-1}^{\left(i,j\right)}-{\hat{m}}_{k|k-1}^{\left(i,j\right)})$$

(37) $${\hat{P}}_{U,k}^{\left(i,j\right)}=(I-{\hat{P}}_{k|k-1}^{\left(i,j\right)}\left(R_{k}+{\hat{P}}_{k|k-1}^{\left(i,j\right)}+H_{k}P_{k|k-1}^{\left(i,j\right)}H_{k}^{T}\right){}^{-1}){\hat{P}}_{k|k-1}^{\left(i,j\right)}$$

### 3.3. Pruning and Merging

In the proposed algorithm, if the multi-Bernoulli joint posterior density at time step k−1 has $M_{k-1}$ hypothesized tracks, then, at time step k, the total number of the updated hypothesized tracks is $M_{k-1}+M_{\gamma ,k}+|Z_{k}|$, and the number of the Gaussian components representing the *i*th measurement-updated track is $(M_{k-1}+M_{\gamma ,k})J_{k|k-1}^{\left(i\right)}$. This indicates that the number of the updated tracks and the Gaussian components representing the multi-Bernoulli posterior density increases with the time step increases, making the computational load very high. Hence, to reduce the computation load, the pruning and merging procedure needs to be implemented after the update step. The detailed pruning and merging procedure used in the proposed algorithm is provided in Algorithm 1.

**Algorithm 1.** Pruning and merging for the proposed algorithm. ***Step 1*.**
*Pruning*    Given the updated multi-target density $\pi_{k}(x_{k},b_{k})={\left\{r_{k}^{\left(i\right)},p_{k}^{\left(i\right)}(x_{k},b_{k})\right\}}_{i=1}^{M_{k}}$ at time step k, and two truncation thresholds $P_{r}$ and $T_{w}$.    Set $I=\{i|r_{k}^{\left(i\right)}\geq P_{r}\}$.    for i=1,⋯,|I|    Set $J^{\left(i\right)}=\{j|w_{k}^{\left(i,j\right)}\geq T_{w}\}$.    end ***Step 2.***
*Merging*    Given a merging threshold $U_{m}$, and a maximum allowable number of Gaussian components $J_{\max}$.    for i=1,⋯,|I|    Set $a^{\left(i\right)}=0$.    if $|J^{\left(i\right)}|>0$    $a^{\left(i\right)}=a^{\left(i\right)}+1$.    $n=\arg\underset{j\in J^{\left(i\right)}}{\max}w_{k}^{\left(i,j\right)}$.    $A=\{j\in J^{\left(i\right)}|\left(m_{k}^{\left(i,j\right)}-m_{k}^{\left(i,n\right)}\right){}^{T}\left(P_{k}^{\left(i,j\right)}\right){}^{-1}(m_{k}^{\left(i,j\right)}-m_{k}^{\left(i,n\right)})\leq U_{m}\}$.    ${\tilde{w}}_{k}^{\left(i,a^{\left(i\right)}\right)}=\sum_{j\in A}w_{k}^{\left(i,j\right)}$.    ${\tilde{m}}_{k}^{\left(i,a^{\left(i\right)}\right)}=\frac{\sum_{j\in A}w_{k}^{\left(i,j\right)}m_{k}^{\left(i,j\right)}}{{\tilde{w}}_{k}^{\left(i,a^{\left(i\right)}\right)}}$.    ${\tilde{P}}_{k}^{\left(i,a^{\left(i\right)}\right)}=\frac{\sum_{j\in A}w_{k}^{\left(i,j\right)}[P_{k}^{\left(i,j\right)}+({\tilde{m}}_{k}^{\left(i,a^{\left(i\right)}\right)}-m_{k}^{\left(i,j\right)})\left({\tilde{m}}_{k}^{\left(i,a^{\left(i\right)}\right)}-m_{k}^{\left(i,j\right)}\right){}^{T}]}{{\tilde{w}}_{k}^{\left(i,a^{\left(i\right)}\right)}}$.    ${\tilde{\hat{m}}}_{k}^{\left(i,a^{\left(i\right)}\right)}=\frac{\sum_{j\in A}{\hat{m}}_{k}^{\left(i,j\right)}}{\left|A\right|}$.    ${\tilde{\hat{P}}}_{k}^{\left(i,a^{\left(i\right)}\right)}=\frac{\sum_{j\in A}{\hat{P}}_{k}^{\left(i,j\right)}}{\left|A\right|}$.    $J^{\left(i\right)}=J^{\left(i\right)}A$.    end    if $a^{\left(i\right)}>J_{\max}$     Discard $a^{\left(i\right)}-J_{\max}$ Gaussian components with lowest weights.    end end ***Step 3*.**
*Output results* Output ${\left\{r_{k}^{\left(i\right)},\left\{{\tilde{w}}_{k}^{\left(i,j\right)},{\tilde{m}}_{k}^{\left(i,j\right)},{\tilde{\hat{m}}}_{k}^{\left(i,j\right)},{\tilde{P}}_{k}^{\left(i,j\right)},{\tilde{\hat{P}}}_{k}^{\left(i,j\right)}\right\}{}_{j=1}^{{\tilde{a}}^{\left(i\right)}}\right\}}_{i\in I}$ with ${\tilde{a}}^{\left(i\right)}=\min(J_{\max,}a^{\left(i\right)})$.

**Remark 1.**  *The notations* |I|*,* $\left|J_{k}^{\left(i\right)}\right|$*, and* |A| *used in Algorithm 1 denote the number of elements in the sets* I*,* $J_{k}^{\left(i\right)}$*, and* A*, respectively.*

In the next section, we analyze the performance of the proposed algorithm compared with the GM-CBMeMBer filter using the Monte Carlo (MC) simulations.

## 4. Simulation Results

To verify the effectiveness of the proposed algorithm, consider a two-dimensional scenario with an unknown and time varying number of targets observed in clutter. The simulation environment was as follows: AMD A8-6600K APU with Radeon HD (tm) Graphics 3.9 GHz, 4 GB DDR3 1600 Memory, Windows 7, and MATLAB R2012a. The sampling period is Δ=1 s. In the dynamic models given in Equations (1) and (2), the kinematical matrices are defined as follows:

$$F_{k-1}=\left[\begin{array}{cccc} 1 & \Delta & 0 & 0\\ 0 & 1 & 0 & 0\\ 0 & 0 & 1 & \Delta \\ 0 & 0 & 0 & 1 \end{array}\right],\;G_{k-1}=\left[\begin{array}{cc} \Delta^{2}/2 & 0\\ \Delta & 0\\ 0 & \Delta^{2}/2\\ 0 & \Delta \end{array}\right],\;Q_{k-1}=\left[\begin{array}{cc} \sigma_{q}^{2} & 0\\ 0 & \sigma_{q}^{2} \end{array}\right],\;H_{k}={\left[\begin{array}{cc} 1 & 0\\ 0 & 0\\ 0 & 1\\ 0 & 0 \end{array}\right]}^{T},\;R_{k}=\left[\begin{array}{cc} \sigma_{v}^{2} & 0\\ 0 & \sigma_{v}^{2} \end{array}\right]$$

The sensor systematic error $b_{k}$ is a first order Gauss–Markov process with transition density function

(38) $$f_{k|k-1}(b_{k}|b_{k-1})=N(b_{k};b_{k-1},B_{k-1})$$

where $B_{k-1}=\text{diag}([\sigma_{b}^{2},\sigma_{b}^{2}])$ and diag(⋅) denotes the diagonal matrix.

The birth process is a multi-Bernoulli RFS with density $\pi_{\gamma ,k}={\left\{(r_{\gamma ,k},p_{\gamma ,k}^{\left(i\right)}(x_{k},b_{k}))\right\}}_{i=1}^{3}$, where $r_{\gamma ,k}=0.03$, and

(39) $$p_{\gamma ,k}^{\left(i\right)}=N(x_{k};m_{\gamma ,k}^{\left(i\right)},P_{\gamma ,k})N(b_{k};{\hat{m}}_{\gamma ,k},{\hat{P}}_{\gamma ,k})\begin{array}{cc} , & i=1,2,3 \end{array}$$

where $m_{\gamma ,k}^{\left(1\right)}={\left[190,2.5,150,-1\right]}^{T}$, $m_{\gamma ,k}^{\left(2\right)}={\left[150,4,180,\;2\right]}^{T}$, $m_{\gamma ,k}^{\left(3\right)}={\left[100,3.5,220,3\right]}^{T}$, $P_{\gamma ,k}=\text{diag}([1,1,\;1,1])$, ${\hat{m}}_{\gamma ,k}={\left[2.5,3\right]}^{T}$, and ${\hat{P}}_{\gamma ,k}=\text{diag}([\sigma_{\gamma }^{2},\sigma_{\gamma }^{2}])$.

The standard deviations $\sigma_{b}$ and $\sigma_{\gamma }$ are known, with $\sigma_{b}=\sigma_{\gamma }=0.1$ m, and the standard deviations of the state and measurement noise are $\sigma_{q}=0.2$ m and $\sigma_{v}=0.5$ m, respectively. The survival probability is $p_{S}=0.99$. The detection probability is $p_{D}=0.98$. The clutter is modeled as a Poisson RFS with the mean r=9 over the region [0,300] × [0,300] m^2^. At each time step, the hypothesized tracks are pruned by using an existence probability threshold of $P_{r}=10^{-3}$, the Gaussian components are pruned and merged by using a weight threshold of $T_{w}=10^{-5}$, and a merging threshold of $U_{m}=4$. The maximum allowable number of Gaussian components is $J_{\max}=100$.

The filtering performance of the proposed algorithm is evaluated by using the optimal subpattern assignment (OSPA) distance [30] defined as

(40) $$d_{OSPA}\left(X,Y\right)={\left(\frac{1}{n}\left(\underset{\pi \in \prod_{n}}{\min}\sum_{i=1}^{m}d^{\left(c\right)}{\left(x_{i},y_{\pi_{i}}\right)}^{p}+c^{p}\left(n-m\right)\right)\right)}^{\frac{1}{p}}$$

where the parameters are set to *p* = 2 and *c* = 50 m in our simulation. To obtain reliable results, 500 Monte Carlo (MC) trials are performed for each algorithm on the same target tracks but with independently generated measurements.

**Figure 1:**
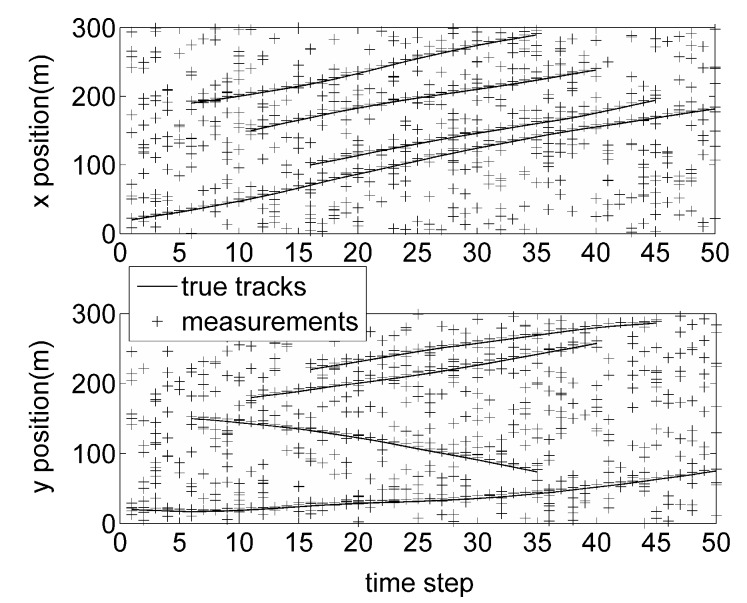
True target tracks and measurements.

In Figure 1, the true target tracks and the cluttered measurements are shown in *x* and *y* coordinates versus time, where the solid lines denote the true target tracks, and the plus signs denote the measurements. Note that there exists one target appearing at time steps 6, 11, and 16, respectively.

**Figure 2:**
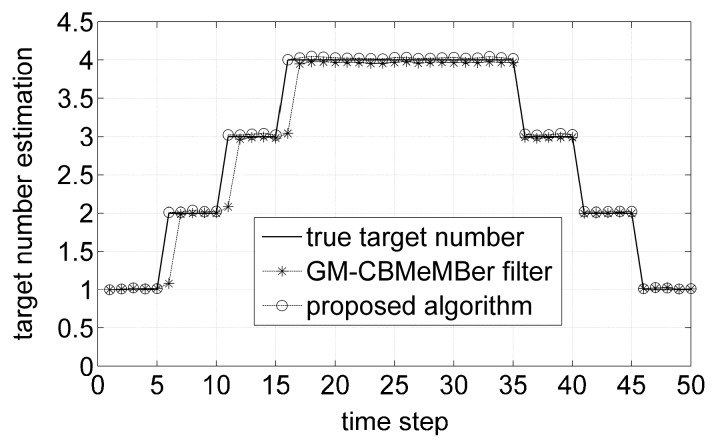
Average target number estimations for different algorithms.

Figure 2 plots the average target number estimations for the GM-CBMeMBer filter and proposed algorithm over 500 MC trials. As seen, at time steps 6, 11, 16, since there is separately one target appearing at those time steps, the proposed algorithm can obtain more reliable number estimations than the GM-CBMeMBer filter. This is due to the fact that the existence probabilities of newborn targets depend on the measurement-updated tracks, which can be computed more accurately by the proposed algorithm.

**Figure 3:**
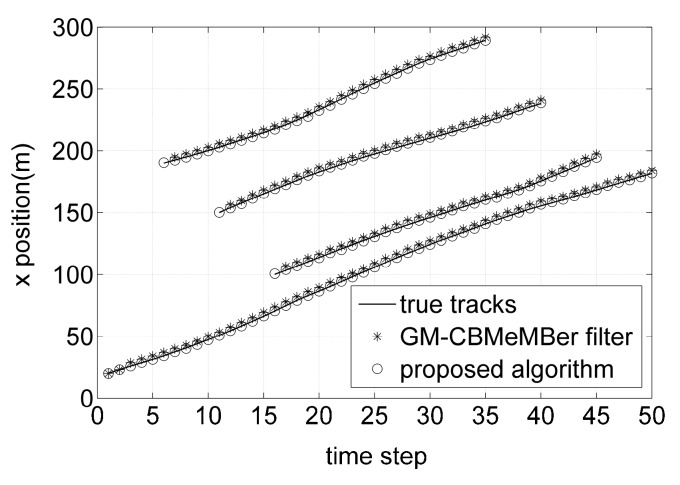
True target tracks and position estimations in *x* coordinate versus time.

**Figure 4:**
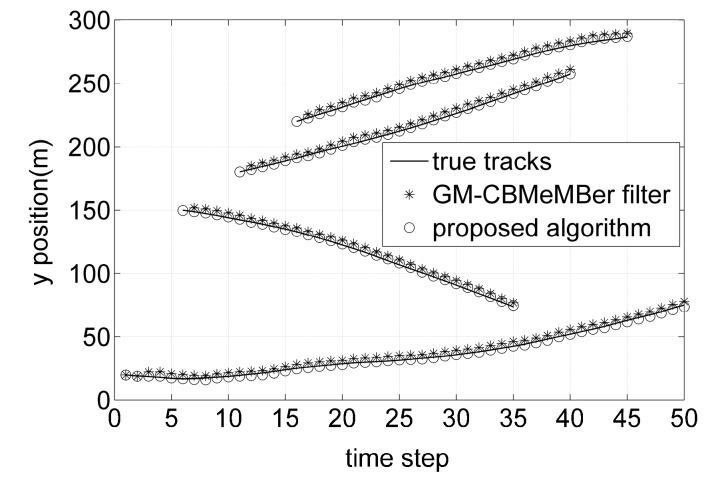
True target tracks and position estimations in *y* coordinate versus time.

Figure 3 and Figure 4 show the individual *x* and *y* coordinates of the true target tracks and the estimated targets against time, respectively. From Figure 3 and Figure 4, it can be seen that the GM-CBMeMBer filter cannot filter out the position estimations of the newborn targets at the time steps where the new targets appear, while the proposed algorithm has no missed position estimations at those time steps. As can be seen by comparing, the target position estimations of the proposed algorithm are closer to the true target tracks than that of the GM-CBMeMBer filter. That is due to the fact that the proposed algorithm can compensate for the systematic errors in sensor measurements during filtering while the GM-CBMeMBer filter cannot.

**Figure 5:**
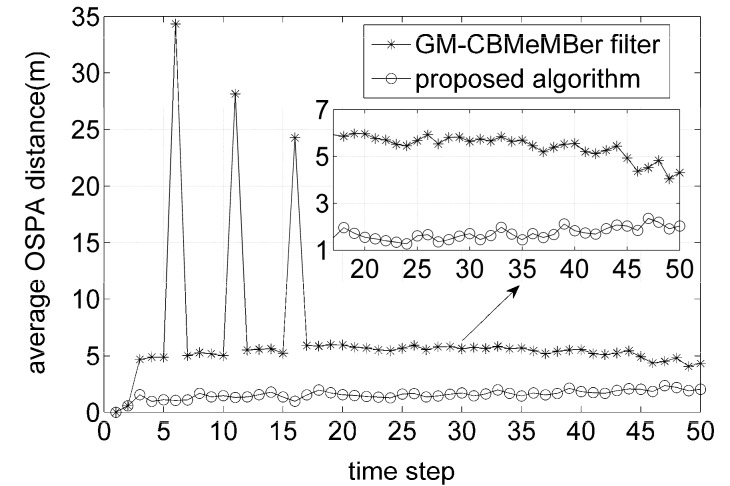
Average optimal subpattern assignment (OSPA) distances for different algorithms.

Figure 5 plots the average OSPA distances for the proposed algorithm and GM-CBMeMBer filter over 500 MC trials. As expected, the results indicate that the proposed algorithm performs better than the GM-CBMeMBer filter throughout the entire filtering process. In addition, it can also be seen that the GM-CBMeMBer filter has three high error peaks at the time steps where the new targets appear.

**Figure 6:**
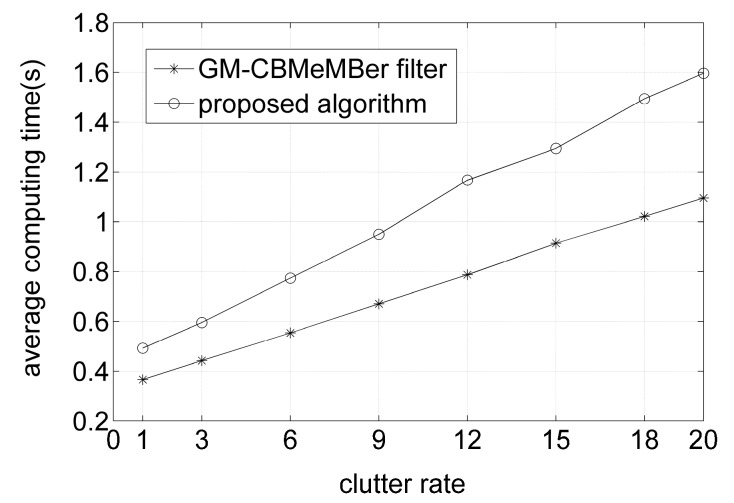
Average computing time for different algorithms versus clutter rate.

**Figure 7:**
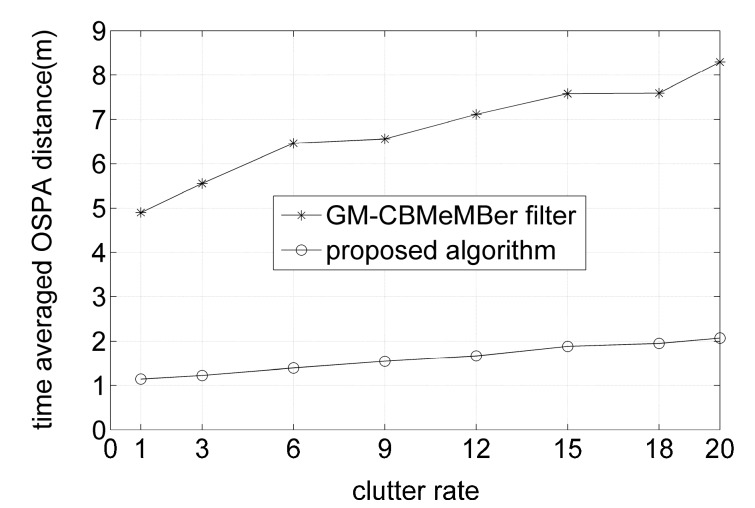
Time-averaged OSPA distances for different algorithms versus clutter rate.

Also, 500 MC trials are performed for both algorithms over varying clutter rates to compare the average performances in terms of the average computing time and time-averaged OSPA distances, with the results shown in Figure 6 and Figure 7, respectively. From Figure 6 and Figure 7, it can be seen that the proposed algorithm needs a bit more time than the GM-CBMeMBer filter to complete one MC trial. However, it outperforms the latter a lot in the aspect of filtering accuracy.

## 5. Conclusions

In this paper, to compensate for the systematic errors in sensor measurements and improve the filtering performance of the CBMeMBer filter, an extended CBMeMBer filter is proposed. Moreover, under the linear Gaussian dynamic and bias measurement models, an analytic implementation of the extended CBMeMBer filter is also proposed by combining the close-form expressions with a pruning and merging procedure to reduce the computation load. Simulation results demonstrate that the proposed algorithm can obtain more reliable target number estimations and achieve better filtering accuracy than the CBMeMBer filter.
